# Evaluating the implementation process of a participatory organizational level occupational health intervention in schools

**DOI:** 10.1186/s12889-016-3869-0

**Published:** 2016-12-01

**Authors:** Roosmarijn M. C. Schelvis, Noortje M. Wiezer, Birgitte M. Blatter, Joost A. G. M. van Genabeek, Karen M. Oude Hengel, Ernst T. Bohlmeijer, Allard J. van der Beek

**Affiliations:** 1 Netherlands Organization for Applied Scientific Research TNO, P.O. Box 3005, NL-2301 DA Leiden, The Netherlands; 2 Body@Work, Research Center on Physical Activity, Work and Health, TNO-VU/VUmc, Amsterdam, The Netherlands; 3 Department of Public and Occupational Health, the EMGO+ Institute for Health and Care Research, VU University Medical Center, P.O. Box 7057, NL-1007 MB Amsterdam, The Netherlands; 4 Department of Psychology, Health and Technology, University of Twente, P. O. Box 217, 7500 AE Enschede, The Netherlands; 5 VeiligheidNL, Overschiestraat 65, NL-1062 XD Amsterdam, The Netherlands

**Keywords:** Process evaluation, Secondary vocational school, Teacher, Mixed methods, Primary prevention

## Abstract

**Background:**

The importance of process evaluations in examining how and why interventions are (un) successful is increasingly recognized. Process evaluations mainly studied the implementation process and the quality of the implementation (fidelity). However, in adopting this approach for participatory organizational level occupational health interventions, important aspects such as context and participants perceptions are missing. Our objective was to systematically describe the implementation process of a participatory organizational level occupational health intervention aimed at reducing work stress and increasing vitality in two schools by applying a framework that covers aspects of the intervention and its implementation as well as the context and participants perceptions.

**Methods:**

A program theory was developed, describing the requirements for successful implementation. Each requirement was operationalized by making use of the framework, covering: initiation, communication, participation, fidelity, reach, communication, satisfaction, management support, targeting, delivery, exposure, culture, conditions, readiness for change and perceptions. The requirements were assessed by quantitative and qualitative data, collected at 12 and 24 months after baseline in both schools (questionnaire and interviews) or continuously (logbooks).

**Results:**

The intervention consisted of a needs assessment phase and a phase of implementing intervention activities. The needs assessment phase was implemented successfully in school A, but not in school B where *participation* and *readiness for change* were insufficient. In the second phase, several intervention activities were implemented at school A, whereas this was only partly the case in school B (*delivery*). In both schools, however, participants felt not involved in the choice of intervention activities (*targeting, participation, support*), resulting in a negative *perception* of and only partial *exposure* to the intervention activities. *Conditions*, *culture* and *events* hindered the implementation of intervention activities in both schools.

**Conclusions:**

The framework helped us to understand why the implementation process was not successful. It is therefore considered of added value for the evaluation of implementation processes in participatory organizational level interventions, foremost because of the context and mental models dimensions. However, less demanding methods for doing detailed process evaluations need to be developed. This can only be done if we know more about the most important process components and this study contributes to that knowledge base.

**Trial registration:**

Netherlands Trial Register NTR3284.

## Background

Work-related stress is highly prevalent among teachers [1, 2] in different countries throughout both the eastern and western developed world [3]. In the Netherlands almost one in five teachers suffers from burnout complaints, compared to one in eight employees in the general working population [4]. Most of the interventions to prevent work-related stress in education aim to increase the resources of the individual to deal with the demands of the job [5–10]. However, these interventions were only partially effective in influencing (dimensions of) burnout [5–7, 9, 10] and well-being [10]. Explanations for this lack of effectiveness could be the level the intervention is aimed at (ie secondary or tertiary prevention), whereas it is proposed that problems should also be addressed at the source (primary prevention) and organizational level to sustainably decrease work-related stress. An effectiveness study of a primary preventive strategy for schools has demonstrated that this approach can indeed help to decrease burnout and to increase efficacy in teachers [11]. However, two meta-analyses of stress management interventions were thus far unable to demonstrate that primary interventions are more effective than secondary or tertiary interventions [12, 13]. This might be due to insufficient or partial implementation of primary, organizational level interventions, which might be explained by the lack of a proper implementation strategy [14–16].

Since it has been suggested that the implementation process can moderate or mediate the potential effects of complex organizational interventions on health or well-being [17], it seems important to study this process. The advantages of a process evaluation are that it helps the interpretation of outcomes [18, 19], sheds light on successes and failures of an intervention [17, 20] and thus shows what parts of the interventions should be improved in replication studies [17]. Lastly, it allows to draw inferences about future applicability in the current setting and about generalizability and transferability to other settings [21–23]. The importance of process evaluations in examining how and why interventions are (un) successful is increasingly recognized [24].

However, a recent review showed that process evaluations of stress management interventions are conducted in an explorative manner mostly, instead of using a theoretical framework [25]. Several models for the evaluation of implementation processes are available though [17, 26–29]. Previous public health intervention studies with comparable outcome measures as in the current study applied the Steckler and Linnan approach [26] to evaluate the implementation process [30, 31]. This process evaluation framework examines context, reach, dose delivered, dose received, fidelity, implementation, recruitment and satisfaction at the individual level. However, we also need to include particular aspects in our process evaluation that seem to be especially relevant for understanding implementation processes in participatory interventions in constantly changing organizations. These aspects are a close examination of the organizational context and participant’s perceptions of the intervention. The first aspect, context, is often narrowly defined as the events that hindered the implementation, whereas the broader organizational context, encompassing also the organization’s culture and capacity to implement the intervention is often also of influence in this type of intervention [32]. The second aspect, the perception of the intervention, may be even more important than actual exposure to the intervention [33, 34], though few studies have actually measured exposure to primary organizational stress interventions, and linked the exposure patterns to outcomes. Studying exposure as well as the organizational context and participant’s perceptions is possible using Nielsen and Randall’s framework [17], which is developed specifically for organizational level occupational health interventions and thus best suited the intervention that is evaluated here. The framework can be applied to quantitatively and qualitatively assess three themes of process components: (i) intervention design and implementation; (ii) context; and (iii) participant’s mental models. To our knowledge, this is one of the first studies in which the framework is applied to evaluate the quality of an implementation process.

The objective of this article is thus to systematically evaluate the implementation process of a primary preventive, participatory, organizational level intervention in two schools, by applying the Nielsen and Randall framework that addresses the intervention, the context and participants’ mental models. The research question is: does the use of this process evaluation framework help us understand why or why not the implementation was successful?

## Methods

The current process evaluation was performed alongside a controlled trial among employees in two secondary vocational education and training (VET) schools, investigating the effectiveness of an intervention on vitality and need for recovery. Detailed information on the methods, procedures and intervention can be found in the protocol article [35]. The project was conducted in two institutions for vocational education in the west (school A) and north (school B) of the Netherlands.

The study protocol and materials were approved by TNO’s Review Committee Participants in Experiments, which is an internal ethics committee that assesses ethical aspects of involving participants in scientific experiments. All participants signed an informed consent before the first measurement.

### Study population

The study population for this process evaluation consisted of teaching and non-teaching employees (ie educational and administrative support staff) and their managers in the intervention departments of both schools (school A, *N* = 150; school B, *N* = 161), including the senior management (ie Executive Board) and two intervention facilitators. The few employees within the intervention departments teaching in general secondary education for adults only were excluded, because they were only administratively part of the intervention departments. In practice, they worked with and belonged to an interdepartmental group of teachers in secondary education for adults.

### The intervention

The intervention under study in the ‘Bottom-up Innovation project’, the Heuristic Method (HM), is a participatory action approach applied at the organizational level. HM consists of two 12-month phases: (i) a phase of needs assessment, and (ii) an implementation phase.

In the first phase intervention activities to increase happy and healthy working are developed in conjunction with relevant stakeholders (ie staff and teachers) under supervision of an intervention facilitator, hereafter referred to as ‘HM facilitator’. The HM facilitator is an expert in organizational change processes, and he or she uses the management’s and employees’ knowledge, skills and perceptions to thoroughly determine what factors hinder and facilitate “healthy and happy working” in the organization. A participatory group of employees (including a staff member) is formed, they assist the HM facilitator and serve as ambassadors of the project. All employees with an interest in the topic of health at work can apply to serve as a participatory group member, and they are then appointed on a first come first serve basis. Tasks for this group are executed within working hours and time spent is compensated. The HM facilitator, assisted by the participatory group, leads three steps to complete the first phase, the needs assessment, by: (i) approximately ten one-hour interviews with typical optimistic and typical critical teachers and staff selected by the participatory group; (ii) a digital questionnaire for all employees; and (iii) group sessions with all teachers within the intervention department, chaired by members of the participatory group. The result of each step in the intervention determines the content of the following step. Reports of each step are written by the HM facilitator. The participatory group approves each report before it is discussed with the management team. After discussion with the management team it is sent and presented to all employees in the intervention group by the HM facilitator. The third and last report, named “advisory report”, is the HM facilitator’s advice to the management team on how to proceed in the next phase.

In the second phase, the implementation phase, the intervention activities are implemented by the middle management team under supervision of the director and supported by senior management (hereafter referred to as ‘the implementers’). HM prescribes that the implementers expand the HM facilitator’s advisory report with an implementation plan, comprising at least a timeframe, a budget and an allocation of roles (eg the role of the participatory group), named “action plan”. Assistance by the HM facilitator can be provided if the implementers have the means to temporarily hire such help.

### Program theory

The program theory is our interpretation of how the intervention would work if implemented as planned.

We assume that by involving an HM facilitator and by involving employees in participatory groups a thorough needs assessment can be conducted and based on that appropriate solutions for the improvement of the working environment can be developed and implemented. In particular we assume that the three steps of the needs assessment phase (ie interviews, questionnaire, group sessions) lead to identify which factors hinder and facilitate “healthy and happy working” in the organization. We further assume that the advisory report based on the comprehensive needs assessment will lead the management to develop an appropriate action plan that addresses the identified problems, describes how and in which time these should be solved, provides the necessary resources and conduct the implementation of these solutions. We also assume that the quick wins, which are part of the action plan, are implemented quickly. Based on these changes we assume that “healthy and happy working” in the organization will improve resulting in the end into improvements in vitality and need for recovery.

#### Requirements

In order to successfully implement this intervention, several requirements need to be met. These requirements are the conditions under which we assume the intervention to work. If the requirements are met, the chance of successful implementation of the intervention increases, and if implementation is successful the chance of finding the hypothesized health effects increases (ie increase in vitality, decrease in need for recovery). This latter part of the study is investigated in an effect evaluation and reported on in another article. All operationalizations of the requirements for successful implementation are described in Table 1 in chronological order of implementation, they are phrased as questions for comprehensibility. The table also states which data source was used to assess whether the requirement was met. Every requirement is assigned to one of the factors in the Nielsen and Randall framework.

**Table 1:** Requirements for successful implementation, their operationalization and data source

*Intervention phase 1: needs assessment*				Source				
*No.*	*Process component*	*Requirement based on program theory*	*Operationalization*	*Quan.*		*Qual.*		
				*Q1*	*Q2*	*Logs*	*I1*	*I2*
1	Initiation^a^	Were senior and middle management committed to the intervention at the start?	• Reasons for middle and senior management to participate			X		X
2	Communication^a^	Was the intervention project communicated to the employees at the start?	• The manner in which the project was communicated to the participants			X		
3	Participation^a^	Was a participatory group formed? Did the employees feel involved in the intervention?	• Composition of group and procedure was in line with protocol • The majority of the participants scored above the cut-off point on the ‘employee involvement’ scale at T1	X		X		X
4	Fidelity^a^	Was intervention phase 1 delivered by HM facilitator according to protocol?	• The extent to which the HM facilitator complied with the needs assessment protocol, according to the facilitator and researcher			X		
5	Reach^a^	Was intervention phase 1 received by majority of the employees?	• Attendance of employees in each step of the needs assessment according to objective attendance rates. The rate expresses the number of those who actually participated in each step out of those who were asked to participate in each step. • The majority of the participants scored above the cut-off point on the ‘exposure’ scale at T1	X		X		
6	Communication^a^	Were results of each step in phase 1 communicated to employees by HM facilitator?	• Percentage of participants who reported to have received a report of intervention step 1 interviews • Percentage of participants who reported to have received a report of intervention step 2 questionnaires • Percentage of participants who reported to have received a report of intervention step 3 group sessions	X				
7	Satisfaction^a^	Were the employees satisfied with intervention phase 1?	• Satisfaction of all employees with (elements of the) needs assessment (ie interviews, questionnaire, group session, advisory report, overall) was moderate (≥6–7.4) or high (≥7.5)^d^	X			X	
8	Middle management support^a^	Was managerial support present at T1 according to management and employees?	• The majority of the participants scored above the cut-off point on the ‘line manager attitudes and actions’ scale at T1 • The managers demonstrated their support of the advisory report	X			X	
9	Readiness for change^c^	Was the majority of the employees at T1 ready for the change?	• The majority of the participants scored above the cut-off point on the ‘readiness for change’ scale at T1	X				
*Intervention phase 2: implementation*								
*No.*	*Process component*	*Requirement based on program theory*	*Operationalization*	*Quan.*		*Qual.*		
				*Q1*	*Q2*	*Logs*	*I1*	*I2*
10	Middle management support^a^	Was an action plan formulated by middle managers based on the advisory report? Were quick wins formulated?	• Middle managers visibly supported the project by designing an action plan including quick wins • The majority of the employees scored above the cut-off point on the ‘line manager attitudes and actions’ scale at T2		X			X
11	Participation^a^	Did employees participate in formulating an action plan?	• The extent to which employees felt responsible for the action plan and the result of implementing the action plan (ie ownership) • The majority of the employees scored above the cut-off point on the ‘employee involvement’ scale at T2 • Formal representatives had a role • Middle managers encouraged active participation by employees		X			X
12	Targeting^a^	Did the action plan target the right problems in the workplace?	• The action plan was applicable to the problems of the workplace • Satisfaction with content action plan was moderate (6.0–7.4) or high (≥7.5)		X			X
13	Senior management support^a^	Did senior management support the action plan?	• Senior managers supported the project throughout • Senior managers allocated the necessary resources			X		X
14	Communication^a^	Was the action plan communicated to the employees? Were small successes celebrated?	• Employees were informed about the action plan and the progress towards its goals • Small successes were celebrated		X	X		
15	Delivery^a^	Was the action plan implemented by middle managers?	• Perceived implementation of the action plan, including quick wins, according to the implementers					X
16	Exposure^a^	Were the employees exposed to implementation of the action plan?	• Perceived implementation of the action plan, including quick wins, according to the employees • The majority of the participants scored above the cut-off point on the ‘exposure to intended intervention’ scale at T2		X			X
17	Culture^b^	Did the organizational culture facilitate the implementation of the action plan?	• Inherent characteristics of the organizational culture that facilitated or impeded the implementation of the action plan					X
18	Conditions^b^	Did the organization have the capacity to implement the action plan?	• The organizational characteristics that affected the (implementation) of the action plan • The organization had the capacity and skills to implement the action plan					X
19	Events^b^	Did no events interfere with the implementation of the action plan?	• Events that occurred and influenced the content or the execution of the action plan			X		X
20	Readiness for change^c^	Was the majority of the employees at T2 ready for the change?	• The majority of the participants scored above the cut-off point on the ‘readiness for change’ scale at T2 • The extent to which employees were ready for change and how this influenced the execution of the action plan		X			X
21	Satisfaction^a^	Were the employees satisfied with intervention phase 2?	• Satisfaction with intervention phase 2 was moderate (6.0–7.4) or high (≥7.5)^d^		X			X
22	Perception^c^	Did implementers and employees perceive the intervention as positive?	• The perception of the action plan was positive • Identify the common grounds and changes in the perception of the action plan					X

*Note. Quan.* quantitative data, ieT1 and T2 questionnaires, *Qual.* qualitative data, ie logs and T1 and T2 interviews, *Q1* questionnaire at T1, *Q2* questionnaire at T2, *Logs* continuous records by logbooks, *I1* interviews at T1, *I2* group interviews at T2

^a^‘intervention’ theme of process evaluation framework

^b^‘context’ theme of process evaluation framework

^c^‘mental models’ theme of process evaluation framework

^d^Satisfaction was rated on a 1–10 scale (1 = very unsatisfied; 10 = very satisfied) and the average satisfaction rate could be classified as poor (*<*6), moderate (≥6 and *<*7.5) or good (≥7.5)

### The process evaluation framework

The framework by Nielsen and Randall [17] for the evaluation of organizational level occupational health interventions was applied. The framework describes three themes of process components that may influence intervention outcomes: intervention, context and mental models. Table 1 lists all operationalizations, in the chronological order of implementing the intervention.

#### Intervention

The ‘intervention’ theme assesses the level of exposure to the intervention by describing (i) the intervention design and implementation, and (ii) the implementation strategy.

The first cluster, intervention design and implementation, was measured by the process components *initiation*, *targeting*, *reach, satisfaction and fidelity. Initiation* was operationalized as the sum of reasons for initiating the intervention (Table 1). This is considered to be an important aspect, since these reasons are likely to influence the initial commitment of all stakeholders [36]. *Targeting* was operationalized as the applicability (ie tailoring) of the intervention to the workplace’s problems (Table 1). This is of importance because every organization is different and therefore requires unique solutions [37] and intervention activities that do not target the right problems are unlikely to bring about the hypothesized effects. The component r*each* was operationalized as the attendance of employees in each step of the needs assessment according to objective and subjective measures (Table 1). Together with *satisfaction* with the intervention and accordance with the protocol (ie *fidelity*), reach demonstrates whether discrepancies exist between the planned intervention and its implementation [26]. This is of importance because an intervention activity cannot be effective if it is not implemented.

The second cluster of process components concerns the roles and behaviors of key stakeholders, also known as the implementation strategy. It comprises: *participation*, *support* of senior and middle management, and *communication*. The component *participation* was operationalized as the extent to which employees actually participated in decision-making (Table 1), and is widely recognized as a precondition for intervention success [38]. Failing to involve employees might lead to a lack of support for intervention activities, dissatisfaction and not targeting the right problems. *Support* of *senior and middle managers* was operationalized as their role throughout the project, including the allocation of necessary resources and possessing relevant skills (Table 1). Senior management support has a direct effect on the actual participation in the intervention [39]. Middle managers are often responsible for implementing the intervention and they are thus also in the position to obstruct or facilitate the change [40]. Finally, the component *communication* was operationalized as the type and quality of the communication about the intervention (Table 1). Successful communication is a way to commit employees to the project by keeping them informed [41] and enabling them to understand the intentions of the implementers [42].

#### Context

The ‘intervention context’ theme comprises the organization’s *culture*, *conditions* (ie the omnibus context; [43]) and *events* (ie the discrete context; [43]) and supposedly moderates or mediates the link between exposure to an intervention and the outcomes. Measuring these three context elements is of importance because they may either facilitate or hinder the implementation of an intervention [17].

The component *culture* was operationalized as those inherent characteristics of a group that facilitate or impede implementation (Table 1). *Conditions* are defined as the capacity of the organization to implement the actions (Table 1). *Events* are occurrences that influenced the content or execution of the actions (Table 1).

#### Participant’s mental models

The theme ‘participant’s mental models of the job and intervention’, comprises re*adiness for change* and (changes in) *perceptions*. This theme concerns all appraisals and perceptions of key stakeholders and how these may drive their behaviors. Comparable to the context, mental models supposedly moderate or mediate the link between exposure to an intervention and the outcomes [17].

*Readiness for change* was operationalized as the extent to which participants are ready for the change the intervention implies (Table 1). It has been argued that interventions can only be effective if participants perceive that problems are present, should be solved and could be solved by the intervention [41].

It was assessed whether *perceptions* of the intervention differed among groups of participants (Table 1). In an intervention context, participants may develop similar ‘models’ to interpret and respond to their work context, including the intervention [44]. If participant’s mental models are not similar this might obstruct implementation, for example because individual have different agendas or see the implemented changes differently.

#### Procedure of applying the framework

Nielsen and Randall [17] proposed a set of questions to help the operationalization of process components into measurable constructs. In three one-hour consensus meetings, four authors (RS, JvG, NW, KOH) adjusted every question to the current study and reached consensus on the data collection tool, the timing of measurement and the implementer perspective (ie middle and senior management,) or participant perspective (ie participatory group, teaching and non-teaching staff; Table 1). Every process component in the framework is assigned to one or more requirements for successful implementation, as is described in the program theory section.

### Data collection

Data were collected by means of questionnaires, interviews and a logbook, from the implementer perspective (ie middle and senior management) or participant perspective (ie participatory group, teaching and non-teaching staff). Process questionnaires, comprising questions on the process components listed in Table 1, were sent out to all participants at 12 months after the start of the study (T1, ie in between intervention phase 1 and 2), and at 24 months after the start of the study (T2). The start of the study is defined by the time the baseline measurements for the outcome evaluations were conducted. Interviews were conducted at T1 and T2. The researcher’s logbook was kept up to date throughout the duration of the study.

#### Questionnaire (measures)

The questionnaires were sent out digitally to all participants and were to be filled out within 6 weeks. Strategies to increase the response rate were: (i) a maximum of three reminders, (ii) the provision of an incentive (ie a book voucher) for those who filled out the complete questionnaire, and (iii) the option to fill out the questionnaire either by telephone communication (school A) or in hard copy (school B), as suggested by the respective participatory groups.

Measures in the questionnaires were a combination of a validated measure and tailor-made, explorative measures. The validated measure is the Intervention Process Measure [45] of which four scales were used: (a) line manager attitudes and actions (eg “My immediate manager has done a lot to involve employees throughout the process”, 7 items), (b) exposure to components of the intended intervention (eg “In this project we openly discuss which traditions or procedures we wish to change and which we wish to keep”, 5 items), (c) employee involvement (eg “I had the opportunity to give my views about the change before it was implemented”, 3 items) and (d) employee readiness for change (eg “I look forward to the changes brought about by the intervention project”, 4 items). Answers were given on a five-point Likert scale from 1 = totally disagree to 5 = totally agree. The scales were all reliable, respective Cronbach’s alphas were: 0.89, 0.80, 0.81, 0.79. In the absence of a clear external criterion, cut-off scores were established by a rule, that is: not more than one item missing per scale and at least two thirds of the items (rounded upwards) has a minimum score of 4. The sum of scores is then divided by the number of items and the cut-off point is determined as the score greater than and not equal to the mean. Respective cut-off points were: (a) 3.14, (b) 2.8, (c) 3 and (d) 3.25. Results are reported as the percentage of participants that scored above the cut-off point. The tailor-made measures were a combination of descriptive yes/no-questions (eg “Are the quick wins implemented?”) and 1 to 10 ratings with higher ratings indicating higher satisfaction (eg “Can you assess the content of the action plan on a scale of 1–10 (where 1 is poor and 10 is excellent)?”. For every question there was an option to elaborate on the response given. Both the validated and explorative measures were tested for comprehensibility by five teachers and one manager at T1 and T2.

#### Interviews

At T1, 17 individual interviews were held (school A, *N* = 11; school B, *N* = 6). At T2, 16 group interviews were held: in school A, 22 participants took part in in total 8 interviews, and in school B, 25 participants took part in in total 8 interviews. All stakeholders were interviewed at T1 and T2: teachers and non-teaching staff, the participatory group, middle and senior management, the two HM facilitators. At T1 interviewees at the participant level were selected based on their high attendance (participation in three or two steps) or low attendance in the needs assessment (participation in one or none steps), to grasp both the perspective of highly exposed participants and low exposed participants.

Interviews were held face-to-face. Seven interviews (six at T1 for school B, one at T2 for school A) were conducted by telephone because of time constraints. All interviews were scheduled for an hour, except for the interview with the participatory group (1.5 h) and senior management (0.5 h).

#### Logbook

In the logbook, the sequence of planned and unplanned events was listed alongside impressions of the principal researcher. In order to do so, the principal researcher was present as an observer at the meetings of the participatory groups in phase 1. For phase 2 the principal researcher based impressions on reading progress reports by the HM facilitator (school A) or holding periodical (telephone) interviews with the school principal (school B).

### Data analysis

Three sources of mixed methods (ie questionnaires, interviews, logbooks) were collected over the course of 24 months to study the implementation components from multiple perspectives, also referred to as methodological triangulation. By comparing and contrasting the perspectives on the implementation components it is possible to arrive at a deeper, wider and more valid understanding of each component than by using only one data source [46]. Two levels of analysis were identified: the implementer perspective and the employee perspective. The implementer perspective comprised two units of analysis (ie middle and senior management) and the employee perspective comprised two units of analysis (ie the participatory group, and teaching and non-teaching staff). Analyses of the implementation components were first conducted within each level and then between levels to identify similarities and discrepancies between the perspectives. Since most elements of the model were covered by the T1 and T2 interviews, we started our analysis with these sources.

Questionnaires were analyzed using the statistical software package SPSS 22 for the closed-ended questions, using descriptive statistics (ie mean, range). Participants had to have filled out T0 to be included in the analyses. Qualitative, open-ended questions were coded manually in the same manner as the interviews.

All interviews were audiotaped and the tapes were transcribed. The first (RS) and second author (NW) separately analyzed the first four transcripts for each school (ie transcripts for interviews with team, director, management and HM facilitator). During a consensus meeting the separate analyses were compared and common themes were identified, in line with the principles of thematic content analysis [47]. This quality procedure served two purposes: i) to ensure consistent and robust coding following the process evaluation framework, and ii) to ensure that every emerging theme was directly supported by data from the interviews or monitoring. After the researchers had agreed on a classification of themes, further analyses were conducted by the first author (RS). Textual segments were marked with codes indicating the process component it was related. The extracted segments were digitally tracked in Microsoft Excel.

The digital and hard copy logbook data were grouped per school to form a chronological list of events, including the impressions of the principal researcher (RS).

## Results

The evaluation of each requirement for successful implementation is described below for school A and B. We distinguish the management perspective (senior and middle management) and the participant perspective (teachers and non-teaching staff, and the participatory group).

### Intervention phase 1: needs assessment

#### Were senior and middle management committed to the intervention at the start? (initiation)

Senior and middle management in both schools decided to participate in the study to solve a given problem in a specific department. In school A this was high sickness absence rates combined with an ageing work population. In school B this was signs of diminishing happiness at work and an ageing work population.

Since the problems were meaningful to the management, we consider the commitment at the start to be high (Table 2).

**Table 2:** Summary of quantitative and qualitative results per process component for school A and B

*Process component*		*Requirement*	*School A*		*School B*	
*Intervention phase 1: needs assessment*			*Quan.*	*Qual.*	*Quan.*	*Qual.*
1	Initiation	Were senior and middle management committed to the intervention at the start?	-	Yes	-	Yes
2	Communication	Was the intervention project communicated to the employees?	-	Yes	-	Yes
3	Participation	Was a participatory group formed?	-	Yes	-	Partly
		Did the employees feel involved in the intervention?	Yes	-	No	-
4	Fidelity	Was intervention phase 1 delivered by HM-facilitator according to protocol?	-	Yes	-	Yes
5	Reach	Was intervention phase 1 received by majority of the employees?	Yes	Partly	No	Partly
6	Communication	Were results of each step in phase 1 communicated to employees by HM-facilitator?	Yes	Yes	Yes	Yes
7	Satisfaction	Were the employees satisfied with intervention phase 1?	Yes	Partly	Yes	Partly
8	Managerial support	Was managerial support present at T1 according to management and employees?	Yes	Yes	Yes	No
9	Readiness for change	Was the majority of the employees at T1 ready for the change?	Yes	-	No	-
*Process component*		*Requirement*	*School A*		*School B*	
*Intervention phase 2: implementation*			*Quan.*	*Qual.*	*Quan.*	*Qual.*
10	Middle management support	Was an action plan formulated by middle managers based on the advisory report?	Yes	Yes	Yes	No
		Were quick wins formulated?	Yes	Yes	No	Partly
11	Participation	Did the employees participate in formulating an action plan?	Yes	No	Partly	No
12	Targeting	Did the action plan target the right problems in the workplace?	Yes	Partly	No	No
13	Senior management support	Did senior management support the action plan?	-	Partly	-	Partly
14	Communication	Was the action plan communicated to the employees?	Yes	Yes	No	Partly
15	Delivery	Was the action plan implemented by middle managers?	-	Yes	-	Partly
16	Exposure	Were the employees exposed to implementation of the action plan?	Partly	Partly	Partly	Partly
17	Culture	Did the organizational culture facilitate the implementation of the action plan?	-	No	-	No
18	Conditions	Did the organization have the capacity to implement the action plan?	-	Partly	-	No
19	Events	Did no events interfere with the implementation of the action plan?	-	No	-	No
20	Readiness for change	Was the majority of the employees at T2 ready for the change?	No	No	Yes	No
21	Satisfaction	Were the employees satisfied with intervention phase 2?	No	-	No	-
22	Perception	Did implementers and employees perceive the intervention as positive?	-	Partly	-	No

*Note. Quan* quantitative data, ie questionnaire at T1 and T2, *Qual* qualitative data, ie continuous records by logs, interviews at T1 and group interviews at T2. (−): this aspect was not assessed quantitatively/qualitatively

#### Was the intervention project communicated to the employees at the start? (communication)

In both schools, the HM facilitator and the participatory group organized a kick-off meeting for all involved, wherein the intervention and accompanying research was explained. The majority of all involved were present at the kick-off, according to logs. All had the possibility to ask questions, and digital slides with information on the project were emailed to all employees of the intervention group afterwards.

We conclude that communication about the intervention project was successful at both schools at the start (Table 2).

#### Was a participatory group formed? And did the employees feel involved in the intervention? (participation)

A participatory group of employees and staff was formed consisting of six and eleven members for School A and school B, respectively. Qualitative data showed a deviation from the intervention protocol in the composition of the participatory group: in school B it comprised five more members than foreseen, since the management wanted all teams to be represented. This deviation hindered the intervention process since the participatory group at school B was too large to function effectively.

Quantitative data showed that in school A, a majority (71.9%) felt involved at this point in the intervention process, while only about one third of employees in school B (34.4%) felt involved (Table 3).

**Table 3:** Summary of quantitative scores for intervention phase 1 (needs assessment) per school

	School A	School B
3. Participation		
% of employees who feel involved in the intervention	71.9% (*n* = 23/32)	34.4% (*n* = 22/64)
5. Reach (% yes)		
Participation in interviews^a^	91.7% (*n* = 11/12)	100% (*n* = 12/12)
Participation in questionnaire^a^	39.3% (*n* = 59/150)	47.8% (*n* = 77/161)
Participation in group sessions^b^	73.3% (*n* = 110/150)	54.0% (*n* = 71/161)
% of participants who feel exposed to the intervention	68.8% (*n* = 22/32)	29.7% (*n* = 28/64)
6. Communication (% yes)		
Received report on interviews?	53.1% (*n* = 17/32)	57.1% (*n* = 40/70)
Received report on questionnaire?	53.1% (*n* = 17/32)	68.6% (*n* = 48/70)
Received advisory report?	93.8% (*n* = 30/32)	65.7% (*n* = 46/70)
7. Satisfaction^c^ (SD)^d^		
Overall	6.5 (1.19) (*n* = 32)	5.9 (1.58) (*n* = 63)
Interviews	7.5 (.57) (*n* = 4)	8.0 (0.89) (*n* = 11)
Questionnaire	7.1 (1.14) (*n* = 26)	7.3 (0.99) (*n* = 55)
Group sessions	6.7 (1.13) (*n* = 26)	6.8 (1.58) (*n* = 38)
Advisory report correct summary of opinions/wishes/needs? (%yes)	64.5% (*n* = 20/31)	65.4% (*n* = 34/52)
8. Middle management support		
% employees who feel supported	68.8% (*n* = 22/32)	53.1% (*n* = 34/64)
9. Readiness for change		
% employees who feel ready for the change	81.3% (*n* = 26/32)	54.7% (*n* = 35/64)

Note. Variables are whenever possible denoted as percentages (cases/n). The n differs per variable due to the operationalization of the variable (eg satisfaction with report interviews only asked if participant reported to have received the report) or due to drop out during the process of filling out the questionnaire

^a^Participation rates in interviews and questionnaire are based on logbook notes

^b^Participation rate in group sessions is based on objective attendance lists

^c^Satisfaction was rated on a 1–10 scale (1 = very unsatisfied; 10 = very satisfied) and the average satisfaction rate was classified as poor (*<*6), moderate (≥6 and *<*7.5) or high (≥7.5)

^d^SD is standard deviation

We conclude that participation was sufficient in school A at this point in time, but not in school B (Table 2).

#### Was intervention phase 1 delivered by HM facilitator according to protocol? (fidelity)

The three major intervention steps (interviews, questionnaire, group sessions) were executed as planned in both schools. Two changes in the execution of these steps were noted in both schools which actually improved the tailoring of the intervention. First, the composition of the step 2 questionnaire: questions were tailored to the specific problems in collaboration with the participatory group, instead of using standardized, validated modules. Second, with regard to the chairing of the group sessions: the participatory groups were supposed to chair the sessions, but no guideline for chairing the meetings was available. Therefore a guideline for chairing the meetings was drafted by the HM-facilitator and researcher, to ensure that information was given to all participants and in the same way (eg information on the aim and duration of the session, anonymity of the data gathered). The researcher observed 11 sessions and concluded that the guideline was used as planned.

These deviations tailored the intervention to the intervention context and thus facilitated the intervention process.

We conclude that the first phase of the intervention was delivered as planned in the protocol at both schools (Table 2).

#### Was intervention phase 1 received by the majority of the employees? (reach)

The majority in school A and B did participate in interviews and group sessions, but not in the questionnaire (Table 3). More than the majority scored above the cut-off point on the ‘exposure’ scale in school A (68.8%), while this was the case for less than a third for employees in school B (29.7%; Table 3).

We conclude that reach in this first phase of the intervention (needs assessment) was only partly successful (Table 2).

#### Were results of each step in phase 1 communicated to employees by HM facilitator? (communication)

The majority of participants did recall to have received the output of each step in the needs assessment, with highest receiving scores in school A for the advisory report (93.8%) and the report on the group sessions in school B (68.6%; Table 3).

Communication halfway through the project was thus considered successful (Table 2).

#### Were the employees satisfied with intervention phase 1? (satisfaction)

All satisfaction scores are shown in Table 3. In school A, the participants were overall moderately satisfied with the implementation of the intervention’s first phase (mean 6.5), whereas this was not the case in school B (mean 5.9).

Satisfaction scores for interviews were however high in both schools (7.5 in school A, 8.0 in school B). Qualitative analyses showed that in the interviews, the profound questioning by the facilitator was valued. Participants in both schools were moderately satisfied with the questionnaire (7.1 in school A, 7.3 in school B). Qualitative analysis of open ended questions in the questionnaire and T1 interviews showed that it was especially valued that ‘the right themes’ were addressed. Again in both schools, participants were moderately satisfied with the group sessions (6.7 in school A, 6.8 in school B). More specifically, most of the participants felt that everyone could freely give his or her opinion (88.9%) and that the chair listened to them (98.6%; results not shown in table). The majority reported a feeling of taking part in potential changes by participating in the group sessions (79.2%) and felt responsible for the outcome of the group session (88.9%; results not shown in table). However, qualitative analysis of open ended questions in the questionnaire showed that some regretted that no solutions were found to the identified problems right away.

In both schools, a majority of participants (64.5% in school A, 65.4% in school B) perceived the HM facilitator’s advisory report based on interviews, questionnaire and group sessions as a correct summary of their opinions, needs and wishes (Table 4 describes the recommendations of the advisory report per school and translation into action plan). However, almost one in three participants who had received the report stated ‘I do not know/No opinion’ to this question at the T1 questionnaire. This could be considered either a sign of dissatisfaction with the report, or a sign of failed ‘reach’.

**Table 4:** Results of the needs assessment and translation into action plan

	*Main content of advisory report delivered by facilitator*	*Main content of action plan* ^*a*^ *constructed by management team*
School A	i) professionalize the teams;	The director, assisted by an HM facilitator, translated the recommendations into an action plan with three goals, six changes and a set of quick wins. GOALS: i) unambiguous management control; ii) competence and professionalism in the teams, and iii) adequate facilities CHANGES: (i) compliance to the workload policy, (ii) structured performance reviews; (iii) a continuous dialogue on the organization of the educational programs; (iv) a leading team activities plan; (v) weekly work meetings; and (vi) personalized competence development plans. QUICK WINS: create adequate facilities by creating a staff room at both locations; place extra walls in some classrooms; place beamers in all class rooms; improve the service by the facilitation services office.
	(ii) professionalize the management;	
	(iii) improve the administrative support and facilities.	
School B	(i) create adequate and effective management control by installing a management team that is approachable, coaching and leading;	The directors of the management team decided to integrate the facilitator’s recommendations in the annual agreements (ie a Management Contract) they made with the Executive Board, instead of writing a separate action plan^b^. A third party coach was attracted to support teams in a previously initiated change towards becoming self-managing. GOALS in the Management Contract were formulated in five headlines: i) strategy; ii) education; iii) personnel; iv) organization; and v) business operations. The most important CHANGE per headline was: i) form alliances with care partners in the region; ii) change the class bound curriculum of two educational programs into more concentrated ‘learning units’; iii) implement performance review policies; iv) make teams function as self-managing units; and v) develop a multi-annual housing plan for the school. No QUICK WINS were formulated.
	(ii) strengthen the power of teams within the school, by letting them develop a team program that can guide their daily work and makes them actually ‘self-managing’;	
	(iii) make administrative procedures more efficient.	

^a^Action plan was termed ‘Management Contract’ in school B

^b^Heading 12 of the results section (targeting) describes that middle managers (other than the two directors) and participants did not see how the advisory report was translated into the Management Contract

We conclude that although satisfaction scores for interviews, questionnaire and group sessions are moderate to high, there are some signs of dissatisfaction (with regard to evaluating the advisory report in both schools and overall satisfaction in school B) which might have been a hindrance factor for some participants at this point in the implementation process (Table 2).

#### Was managerial support present at T1 according to management and employees? (middle management support)

In both schools, the majority of the participants perceived managerial support with regard to the intervention (68.8% in school A, 53.1% in school B) as was demonstrated by quantitative analyses (Table 3).

In school A, the managers were shocked by the problem definition in the advisory report, leading to a state of apathy which slowed down initial actions. As will be outlined below, support was manifested as soon as the shock was descended.

The directors in school B were not satisfied with the advisory report, which they considered to be too focused on the leading role of the management, whereas the school tried to make the transition towards self-managing teams.

In sum, managerial support was present in school A at T1 and only partly present in school B (Table 2).

#### Was the majority of the employees at T1 ready for the change? (readiness for change)

Quantitative analyses showed that the majority of the participants in both schools was indeed ready for the change (Table 2). Scores were higher in school A (81.3%) than in school B (54.7%; Table 3).

### Intervention phase 2: implementation

#### Was an action plan formulated by middle managers based on the advisory report? And were quick wins formulated? (middle management support)

With the help of an HM facilitator, the management team of school A developed an action plan that included quick wins (Table 4). In school B, the directors of the management team decided to integrate the recommendations in the advisory report with the annual agreements made with the Executive Board, instead of writing a separate action plan. The annual agreements were named ‘Management Contract’ (Table 4) and did not comprise a timeframe, a budget and an allocation of roles. The directors did identify several quick wins, however these were not stated in the Management Contract.

A majority of employees perceived that managerial support for the action plan or its equivalent was present at T2 (57.1% in school A, 51.1% in school B, Table 5).

**Table 5:** Summary of quantitative scores for intervention phase 2 (implementation phase) per school

	School A	School B
10. Middle management support (% employees that perceives managerial support for action plan^a^)	57.1% (*n* = 32/56)	51.1% (*n* = 23/45)
11. Participation (% yes)		
I feel responsible for the implementation of the action plan^*a*^	73.2% (*n* = 41/56)	76.1% (*n* = 35/46)
I feel responsible for the content of the action plan^*a*^	71.4% (*n* = 40/56)	76.1% (*n* = 35/46)
*Employee involvement* (% employees who feel involved in action plan)	55.4% (*n* = 31/56)	37.0% (*n* = 17/46)
12. Targeting (SD)^b^		
Satisfaction with content of the action plan^a^	6.5 (1.31) (*n* = 61)	5.4 (1.96) (*n* = 49)
14. Communication (% yes)		
*Are you informed about the progress in the action plan* ^*a*^ *?*	86.7% (*n* = 52/60)	38.3% (*n* = 18/47)
*How were you informed (more answers possible)?*		
Via information meetings organized by the management team	75% (*n* = 39/52)	61.1% (*n* = 11/18)
Via work meetings	44.2% (*n* = 23/52)	44.4% (*n* = 8/18)
Via the news letter	40.4% (*n* = 21/52)	38.9% (*n* = 7/18)
In another way	7.7% (*n* = 4/52)	16.7% (*n* = 3/18)
16. Exposure (% yes)		
*Have you been informed on the existence of the action plan* ^*a*^ *?*	100% (*n* = 61/61)	87.8% (*n* = 43/49)
*Are you familiar with the goals of the action plan* ^*a*^ *?*		
unambiguous management control teams	83.6% (*n* = 51/61)	-
competence and professionalism in the teams	90.2% (*n* = 55/61)	-
adequate facilities	90.2% (*n* = 55/61)	-
make teams the central executive units	-	89.8% (*n* = 44/49)
adequate and effective management control	-	67.3% (*n* = 33/49)
*Have you seen changes towards these goals:*		
unambiguous management control teams	43.3% (*n* = 26/60)	-
competence and professionalism in the teams	43.3% (*n* = 26/60)	-
adequate facilities (quick wins)	83.6% (*n* = 51/61)	-
make teams the central executive units	-	59.6% (*n* = 28/47)
adequate and effective management control	-	25.5% (*n* = 12/47)
*Are the quick wins* ^*c*^ *celebrated as a success?*	67.2% (*n* = 41/61)	-
*Did you notice a change in:*		
a dialogue on the organization of education	78.3% (*n* = 47/60)	-
performance reviews	76.7% (*n* = 46/60)	-
team activities plan	58.3% (*n* = 35/60)	-
work meetings	56.7% (*n* = 34/60)	-
workload policy	45% (*n* = 27/60)	-
competence development plan	28.3% (*n* = 17/60)	-
teams develop a team- and educational program conform the regulations	-	66.0% (*n* = 31/47)
a better and larger management team	-	57.4% (*n* = 27/47)
management sets guiding standards	-	31.9% (*n* = 15/47)
management is permanently accessible	-	36.2% (*n* = 17/47)
management coaches teachers in educational leadership	-	19.1% (*n* = 9/47)
management eliminates ‘cumbersome’ administrative procedures	-	8.5% (*n* = 4/47)
% of participants that feels exposed to the intervention	56.9% (*n* = 33/58)	39.1% (*n* = 18/46)
20. Readiness for change (% employees who feel ready for the change)	46.4% (*n* = 26/56)	54.3% (*n* = 25/46)
21. Satisfaction (SD)^b^ with implementation of the action plan/advisory report	5.7 (1.4) (*n* = 58)	4.4 (1.7) (*n* = 46)

^a^ Since no action plan was constructed in school B, the question concerned the advisory report

^b^ SD is standard deviation. Being content with action plan was rated on a 1–10 scale (1 = very unsatisfied; 10 = very satisfied) and the average satisfaction rate could be classified as poor (*<*6), moderate (≥6 and *<*7.5) or high (≥7.5)

^c^ The quick wins are the ‘adequate facilities’ in school A

In conclusion: in school A an action plan including quick wins was formulated, whereas no action plan and no quick wins were formulated in school B. In both schools, the majority of employees perceived that managerial support for the action plan was present (Table 2).

#### Did employees participate in formulating an action plan? (participation)

In both schools, the majority of participants felt responsible for development and execution of the action plan (Table 5), suggesting that ownership of the intervention was high.

In school A, quantitative data showed that the majority of employees felt involved in the action plan (55.0%; Table 5). However, qualitative data revealed that employees on the lowest level of the schools hierarchy (eg administrative staff), saw the fewest possibilities to participate, suggesting that managers did not sufficiently encourage employees at all levels to participate. Furthermore, the contact with formal employee representatives (ie Works Council) in formulating the action plan was poor. Lastly, halfway through the intervention period the composition of the participatory group changed and it functioned as a feedback group for the director rather than a group that joined decision making.

In school B, employees perceived that there was no actual participation of employees in the second phase of the intervention (Table 5). The qualitative data showed that the implementers agreed with this perception of employees, because they did not undertake efforts to involve employees in formulating the Management Contract. Contact between implementers and formal employee representatives (ie Works Council) was scarce.

We can conclude that both implementers and employees showed high ownership of the intervention, but procedures to ensure participation were insufficient (school A) or lacking (school B) and therefore participation in formulating the action plan was low in both schools (Table 2).

#### Did the action plan target the right problems in the workplace? (targeting)

Qualitative analysis showed that, according to the implementers in school A, the action plan suited the problems in the workplace. The middle management team reported that the choice for the activities in the action plan was obvious, since it were all things that had to be done already to meet requirements from the school, Inspectorate or Ministry of Education. The participants partially agreed with this view. Teachers and the participatory group were disappointed that they could not participate in the translation of the advisory report into an action plan. To them, the link between their problems and the actions taken was unclear. Quantitative analysis showed that satisfaction with the content of the action plan was moderate in school A (6.5; Table 5).

In school B, qualitative data showed that the participants were unable, even with help from members of the Works Council, to discover how the advisory report was translated into the Management Contract. Quantitative analysis showed that satisfaction with the content of the action plan was poor in school B (5.4; Table 5).

In sum, the implementers in school A and B thought action plans targeted the actual problems in the workplace, whereas employees in school A only partly agreed to that and employees in school B did not agree to that (Table 2).

#### Did senior management support the action plan? (senior management support)

In school A, the senior management (ie Executive Board) financially supported the project throughout. However, most teachers perceived the Executive Board as obstructing certain desired innovations (eg wireless Internet), whilst the participatory group noted that the Board provided constant support. This latter view was shared by all implementers.

In school B, senior management supported the project at first, but decided not to financially support the second phase of the intervention by hiring an HM facilitator again. The Board rather advised to make use of the services of an internal advisor. The Executive Board agreed with the management team that the advisory report’s recommendations were aligned with the Management Contract. And, in retrospect, the Executive Board doubted the decision to take part in the intervention project at all, because the intervention interfered with an ongoing transition towards self-managing teams.

Thus, in both schools senior management was partly supportive (Table 2).

#### Was the action plan communicated to the employees? (communication)

In school A, the majority of the participants was informed about the progress towards the action plan’s goals (86.7%), especially via information meetings organized by the management team (75%; Table 5). Qualitative data showed that the implementers thought their communication strategy was coherent with their implementation strategy, whereas the participants thought communication of actions or results was foremost (and for unclear reasons) initiated too late. Small successes in the implementation of actions were celebrated (eg cake on the opening day of the staff room).

In school B, a minority of the participants was informed about the progress towards the advisory report’s goals (38.3%; Table 5). The participants named several examples of malfunctioning communication due to indecisiveness by the management or due to untimely planning. The management team acknowledged that communication was a concern and that reflections on decisions or planning were lacking.

We conclude that communication of the action plan and its progress was well-organized and effective at the school that formulated the action plan (school A), but less well and effective at the school that did not formulate an action plan (school B; Table 2).

#### Was the action plan implemented by middle managers? (delivery)

In school A, the implementers noted that several changes had been made. They wanted to make the changes consecutively and not parallel, so they started with three of the six intervention activities of the action plan (ie ‘a dialogue on the organization of education’, ‘performance reviews’ and ‘team activities plan’). The other three intervention activities were due later. Some middle managers pointed to the importance of proper and timely implementation, especially of quick wins. For example, at one of the two school locations the staffroom was placed amidst the class rooms and nicely decorated, whereas this was not the case at the other location. This led to corresponding differences in satisfaction with the quick wins per location.

In school B, some of the middle managers perceived little or no changes as a result of the intervention. They found possible progress hard to determine, because of the alignment of the recommendations in the advisory report with the management contract. Senior management and directors reported beginning, yet unstable positive changes towards the goals in the management contract. Furthermore, the identified quick wins were not followed-up in the management contract.

In sum, the implementers in school A perceived the action plan as implemented, whereas this was not fully the case in school B (Table 2).

#### Were the employees exposed to implementation of the action plan? (exposure)

The majority of the participants in school A found that the quick wins were implemented (83.6%, Table 5). However, execution of these quick wins was considered not timely and satisfaction with the way quick wins were implemented differed at the two school locations. Still, two third of the participants (67%) thought the quick wins were celebrated as a success (Table 5). In school B were no quick wins formulated.

In school A, all participants reported to have been informed about the existence of the action plan (Table 5). The majority of the participants was familiar with the other two goals of the plan (83.6; 90.2%) and some changes towards these goals were noted (43.3; 43.3%; Table 5). The majority (56.9%) did feel exposed to the intervention in general. Changes were observed by 28.3-78.3% of the employees, depending on the intervention activity in the action plan (Table 5). The three intervention activities that were delivered according to the implementers indeed had the highest scores (‘a dialogue on the organization of education’, ‘performance reviews’ and ‘team activities plan’). Qualitative data showed however that participants judged the changes in general to be minor, some of the improvements were considered already commonplace before the intervention or not really an improvement after all.

In school B, a majority of the participants (87.8%) reported to have been informed about the existence of the advisory report (Table 5). Most of them (67.3-89.8%) were familiar with the two goals of the advisory report and some changes were noted towards these goals (25.5 and 59.6%; Table 5). A minority (39.1%) did feel exposed to the intervention in general. Percentages of perceived implementation of the recommendations in the advisory report were generally lower than in school A: improvements were observed by 8.5-66.0% of the participants, depending on the intervention activity (Table 5). Qualitative data also showed that the participants perceived little or no changes as a result of the intervention. They also reported that possible progress was hard to determine, because of the alignment of the recommendations in the advisory report with the management contract.

Overall, employees in both schools were aware of the action plan (or management contract) and its content. In school A more intervention activities were perceived as implemented than in school B, but the actual changes were perceived as minor in both schools (Table 2).

#### Did the organizational culture facilitate the implementation of the action plan? (culture)

In school A, implementers and employees reported that the intervention department’s culture was characterized by a distant relation between management and ‘shop floor’, which hindered implementation because of lacking mutual trust.

In school B, both the implementers and participants pointed to the ‘collective history’ of this school as a reason for lacking implementation. According to the implementers, teachers demonstrated either some sort of learned helplessness, or high levels of independency, which hindered the implementation process. Some of the employees felt that over the years formerly friendly hierarchical relations had developed into business relations, constructing ‘a culture of fear’, and this belief also hindered the implementation process.

In short, the culture was a hindrance in implementing the intervention in both schools (Table 2).

#### Did the organization have the capacity to implement the action plan? (conditions)

In school A, an HM facilitator was temporarily hired to facilitate the second phase of the intervention. The implementers considered the intervention time and energy consuming, whereas the investment was only perceived as ‘returned’ if progress was notable for all involved. The participants perceived the structured, target-driven way of implementing the intervention activities in the second phase not suited to the school structure that valued autonomy of the individual teacher.

In school B senior management insisted that the middle managers received coaching in implementing the management contract, because without the coaching, they had too little confidence that the management contract would be realized. The management team changed during the implementation phase: only two of the seven members stayed in position, including only one of the two directors. The two directors were responsible for executing the management contract and this hindered the implementation process.

In sum, school A had more capacities to implement the action plan than school B (Table 2).

#### Did no events interfere with the implementation of the action plan? (events)

The Ministry of Education announced a structural change for the secondary vocational education and training sector, implying an intensification of classroom-bound lessons from 850 to 1000 h per year. In School A, an intensification trajectory was developed to redesign the curriculum (in school B, no preparations were made to make this change). At the same time in school A, fewer students enrolled and therefore inflow of tuition fees decreased. Temporary contracts had to be ended. In parallel, the Executive Board decided that the 7 week summer holiday had to be reduced to 6 weeks. All events hindered the implementation of intervention activities, foremost because not every activity could be maintained in these changing circumstances.

In school B, two curriculums and their 34 respective teachers were positioned in another department within the greater college. Secondly, teams were reorganized and their composition changed. Thirdly, only two of the seven earlier members of the management team were still there at the end of the implementation phase. Fourthly, a third party advisor, other than the consultant appointed by the Board, came and went. Fifthly, structural changes in the Dutch healthcare sector were planned, which influenced the composition of the educational curriculum. All events hindered implementation, foremost because they interfered with the planned changes in the management contract.

Thus, at both schools unexpected events negatively interfered with the implementation process over the course of 24 months (Table 2).

#### Was the majority of the employees at T2 ready for the change? (readiness for change)

Qualitative analysis of school A showed that, the participants were disappointed in the lack of timely progress towards the action plan’s goals, after the positively perceived first phase of the intervention. During the implementation phase the organization functioned top-down instead of bottom-up, and the actions in the action plan were all considered management instruments, leading to skepticism on the shop floor. The quantitative analysis confirmed this, since a minority scored above the cut-off point on the readiness for change scale (46.4%, Table 5).

In school B, qualitative data showed that the implementers and the participants really wanted positive changes, but both did not know how to contribute to this change process and the intervention did not support this sufficiently. The middle and senior managers reported beginning, yet unstable positive changes in the way managers and employees interacted in general and about the Management Contract. This seemed to be reflected in the quantitative analysis of readiness for change in participants: a majority was ready for change at T2 (54.3%, Table 5).

In sum, readiness for change was insufficient at T2 in school A due to disappointment in the result of the intervention activities, whereas in school B the majority seemed to be ready for the change (Table 2).

#### Were the employees satisfied with intervention phase 2? (satisfaction)

In both schools, overall satisfaction with the implementation of the action plan was poor (Table 5). The low satisfaction scores are considered the result of the sum of program requirements that were not met (Table 2).

#### Did implementers and employees perceive the intervention as positive? (perception)

In school A, the implementers were shocked by the problem definition in the advisory report, leading to a state of apathy which slowed down initial actions. Implementers noted that the needs assessment phase led to participants’ high expectancies of quick changes, whereas the action plan had to be drafted and actions implemented, which was time-consuming. And exactly this time-consuming process proved the to the participants that the organizations’ problems were still not solved.

In retrospect, the implementers in school B expressed their doubts about the whole intervention project, since the added value compared to all ongoing measures was unclear to them. The participants were disappointed in the intervention project since no progress was observed.

In summary, the intervention was not fully perceived as positive in both schools (Table 2).

## Discussion

Our objective was to systematically evaluate the quality of the implementation process of a primary intervention for workplace mental health, by using a theoretical framework. The research question was whether the use of this process evaluation framework helps us to understand why the implementation was or was not successful.

### Main findings

We have demonstrated that the Nielsen and Randall process evaluation framework indeed helps us to understand why the implementation was not successful and before discussing the implications, we summarize the results (Table 2). In school A, all intervention requirements for successful implementation of intervention phase 1 were met. However, it seems that not all employees were reached (no.5) and were only partly satisfied (no.7). In school B, two intervention requirements for successful implementation were not met: employees did not feel involved in the intervention (no. 3) and only a minority was ready for the change (no. 9). Furthermore, as in school A, reach (no. 5) and satisfaction (no. 7) were only partly met and in addition, managerial support (no. 8) was confirmed in the quantitative data but not in the qualitative in school B.

In the second phase in both schools most requirements were not met, except for middle management support (no. 10), communication (no. 14) and delivery (no. 15) in school A. Some requirements were partly met, that is senior manager support (no. 13) and exposure (no. 16) for both schools. Mixed findings, wherein results from quantitative and qualitative analysis pointed in a different direction (yes/no), were found for the requirements participation (no. 11) in school A, and middle manager support (no. 10) and readiness for change (no. 20) in school B.

### Findings in context of the literature

The results resemble the findings reported in the literature on the implementation of participatory action approaches. That is, it has been convincingly demonstrated that partial implementation can have detrimental effects on commitment to the intervention [48]. Partial implementation might be due to a range of factors that all were present to some extent in the current study: a changing organizational context, low ownership of stakeholders and flaws in the intervention design [48].

Managing the perceptions of the intervention process seems of utmost importance for successful implementation of the intervention. In our study, participants who felt not involved in the intervention did not perceive changes, as has been demonstrated before [49]. The notion that the perception of the change may be even more important than actual exposure to the change [33, 34] was underlined by this finding. The even more explicit statement that “more harm can be done by disappointing expectations than by not conducting an intervention” [50] was also confirmed by the results of this process evaluation.

Furthermore, the organizational culture and conditions were suboptimal for implementing an intervention in both schools. The participating schools chose the intervention groups instead of random allocating them to an intervention or control condition, and trust between managers and employees in the appointed intervention groups turned out to be fragile at both schools. The earliest participatory action research studies in occupational health already showed that involvement in the intervention only led to increased participation in decision making when cooperative relations were present (eg [32]). The intervention did not succeed entirely to overcome the suboptimal preconditions for implementation. Organizational culture and the capacity to implement the intervention (conditions) are requirements for successful implementation that can be assessed before the start of the intervention, so to decide whether the intervention project should be initiated at all.

### Implications for research

To our knowledge, this is one of the first organizational level participatory interventions evaluated by using the Nielsen and Randall framework [17]. In our experience the framework is a comprehensive tool for designing a process evaluation and accompanying data collection. With this framework, we captured context factors and perceptions that are known to be of importance in this type of intervention. For example, because of the “participant’s mental models” dimension we were able to demonstrate that the partial implementation was due to disappointment in the results of phase 2, which probably reduced readiness for change and definitely increased negative perceptions of the intervention and the organization.

Process evaluation and frameworks for process evaluation are in a dynamic development process. We think process evaluation should be based on 1) a combination of quantitative and qualitative data, and 2) the most appropriate model which is available at the time when the evaluation is done. First, adding qualitative measures increases the chance of grasping the story behind the numbers and we demonstrated that this story might point in a different direction for middle management support (10; school B), participation (11; school A) and readiness for change (20; school B). Second, future process evaluations might use other frameworks building on experiences with existing frameworks. We highlight three recent frameworks that might be helpful, in addition to the one applied and the ones already mentioned in the introduction. The framework by Fridrich and colleagues [51], which suggests to view the entire intervention process as a continuous change and thus suggests to blur the line between process and outcome evaluation by distinguishing between proximate, intermediate and distal outcomes of interventions. Or the model by Von Thiele Schwarz and colleagues [52], that integrates intervention design and intervention evaluation, because the collected data is used to adapt intervention activities. Or otherwise the generic model by Damschröder and colleagues [53], that was based on a combination of existing frameworks and contains several potentially relevant domains for the study of implementation processes (ie the intervention, the outer setting, the inner setting, individual characteristics, the process).

In the current evaluation, the initiation and needs assessment phase plays a large role, and even though these are part of the Nielsen and Randall framework we had to develop a number of evaluation aspects for the initiation and needs assessment phase, since they were not listed in the model (eg satisfaction). And while the framework is useful for designing the process evaluation and data collection, it is less useful in describing the results. Foremost because the framework does not provide a strategy to relate the fragmented process components to each other, it is unclear which process components are most important. This leaves room for the individual researcher to interpret or weigh the components at her discretion, and that can be a risk for the replicability of process evaluations and the generalizability of their conclusions. There are too few detailed process evaluations that would make testing these process components possible, so we encourage researchers to conduct more detailed process evaluations. We recommend to develop a program theory that lists all requirements for successful implementation of the intervention. Then use the framework to operationalize all requirements for successful implementation, assess whether requirements are met by comparing the actual implementation to the requirements in the program theory [54].

There is also a downside to the comprehensiveness of the framework. Extensive data collection needs to be carried out to cover all headings in the framework, especially if one is interested in perceptions of different stakeholders and changes over time. This can be difficult and is time-consuming. Moreover, it can be challenging to present the huge amount of data in an attractive but concise manner. However, it seems important to continue conducting detailed process evaluation studies in order to advance our knowledge about what is needed to make organizational level and participatory workplace interventions work. Less demanding methods for doing detailed process evaluations need to be developed, but we can only do that if we know more about process evaluations, and this study contributes to that knowledge base.

### Implications for practice

The HM intervention was developed by a Dutch consultancy firm and had been applied over a hundred times in the last decade mainly in public organizations, prior to evaluation within the current trial. We, in the role of independent researchers, expected the intervention to do well in a trial after such extensive piloting and adjusting. We also expected positive results because the intervention encompassed a participatory action approach, which provides an implementation strategy [38, 55, 56].

However, we encountered two flaws in the intervention design. Firstly, the protocol did not support the transition from HM facilitator as a primary implementer in phase 1 to the management team in phase 2. Neither did the intervention protocol provide sufficient guidance on what to do if the management did not take account of the advisory report. As a result, the intervention process stagnated at first due to resistance to the advisory report among the management teams in both schools. The intervention ought to be revised at this point, for example by specifying the intervention protocol and managing the schools’ expectations of the intervention better and from the beginning. Secondly, the intervention ought to facilitate fast implementation of quick wins, so to fulfill expectations and make optimal use of the readiness for change resulting from phase 1. There seems to be a ‘window of opportunity’ wherein actual changes will be perceived. If this window is missed, it will be hard to successfully implement the intervention.

### Strengths and limitations

A strength of the current study is the use of a theoretical framework, which is not yet commonplace according to a review of process evaluations of stress management interventions [25]. The long term follow-up is also an apparent strength (eg [57]), which makes the findings worthwhile. The mixed methods design dealt effectively with both recall bias and common method bias, since both objective and subjective measures were combined.

A limitation of the current study is the rather low response rate we encountered at first follow-up, despite all efforts to increase the response. This is a common problem in intervention research (with intensive process evaluation) [58]. Since the first phase of the intervention consisted of a comprehensive needs assessment, which drew on a number of research methods (ie interviews, questionnaire, group sessions), participants supposedly became “research-tired” when receiving the T1 and later on T2 questionnaire. Furthermore, 34 employees in school B were relocated outside the intervention department shortly prior to T1, which might have hampered their motivation to fill out the questionnaire. The low response to the questionnaires limits the generalizability of the results somewhat. However, since different data sources were triangulated while studying the same phenomenon (ie different sources, different type, different measurement moments), we were able to cross verify our findings, which still makes them worthwhile.

Finally, a shortcoming in the evaluation of organizational level occupational health interventions is that process and effect data are often not combined. This comprehensive process evaluation did not integrate data either. However, we can formulate more specific hypotheses about the intervention effects than we could have done without this process evaluation: implementation of the intervention’s first phase was more successful than implementation of the intervention’s second phase in both schools. We thus expect to find an effect of the intervention’s first phase on occupational self-efficacy, but no effect or even a negative effect on organizational efficacy, job characteristics and health outcomes as a result of the second phase.

## Conclusion

The Nielsen and Randall process evaluation framework helped us to understand why the implementation process of an intervention was not successful and it is therefore considered of added value for the evaluation of implementation processes in participatory organizational level interventions. However, the framework requires collecting a large amount of qualitative and quantitative data and extensive data analysis. Less demanding methods for doing detailed process evaluations need to be developed. This can only be done if we know what are the most important process components and this study contributes to that knowledge base.

## Abbreviations

HM: Heuristic Method, the trademark name of the intervention under study
T0: Baseline measurement
T1: First follow-up measurement
T2: Second follow-up measurement
